# Age and Gender Difference in the Association of Metabolic Syndrome and Peripheral Artery Disease Among Patients With Type 2 Diabetes Mellitus

**DOI:** 10.1155/ijvm/5572344

**Published:** 2025-03-10

**Authors:** Xiaotong Feng, Yongsong Xu, Lin Zhu, Kun Li, Lin Mao, Huan Dong, Dong Zhao, Jing Ke

**Affiliations:** ^1^Center for Endocrine Metabolism and Immune Diseases, Beijing Luhe Hospital, Capital Medical University, Beijing, China; ^2^Beijing Key Laboratory of Diabetes Research and Care, Beijing, China

**Keywords:** peripheral artery disease, metabolic syndrome, Type 2 diabetes mellitus

## Abstract

**Purpose:** Our study is aimed at exploring the association between peripheral artery disease (PAD) and metabolic syndrome (MetS) across different age and gender groups among patients with Type 2 diabetes mellitus (T2DM).

**Patients and Methods:** A total of 3638 patients with T2DM were enrolled in the study, including 281 patients with PAD and 3357 patients without PAD. Demographic data and anthropometric measurements, such as height, weight, and waist circumference, were collected. Laboratory tests and ankle–brachial index (ABI) assessment were also conducted. Multiple logistic regression was used to evaluate the relationship between PAD and the number of MetS components across different age and gender groups.

**Results:** After adjusting for potential confounding factors, our results indicated that the ORs for the presence of PAD increased progressively with the number of MetS components. Stratified analysis showed that this effect was particularly pronounced in younger patients (aged < 40 years) and older patients (aged ≥ 60 years), where the risk of PAD rose with an increasing number of MetS components. Furthermore, the positive association between the number of MetS components and PAD presence was significantly stronger in females.

**Conclusions:** In summary, our findings suggest that the risk of PAD is positively correlated with the number of MetS components in patients with T2DM, especially among younger patients and older patients. Additionally, the positive association between the number of MetS components and the presence of PAD was significantly more evident in female patients.

## 1. Introduction

Peripheral artery disease (PAD) is characterized by the narrowing or blockage of arteries, ranging from the aortoiliac segment to the pedal arteries. The global prevalence of PAD was 5.6% (236 million adults) in 2015, with the number of cases increasing by approximately 45% from 2000 (163.6 million adults) to 2015 [1]. Numerous studies have shown that PAD is a strong predictor of future myocardial infarction and stroke. Moreover, PAD is the third leading cause of atherosclerotic morbidity, following coronary heart disease and stroke [2]. The development of PAD is multifactorial, with risk factors including smoking, diabetes, high-fasting serum cholesterol level, hypertension, and chronic kidney disease [3–5].

Metabolic syndrome (MetS) is characterized by abdominal obesity, insulin resistance, hypertension, and hyperlipidemia. It was first formally defined through a working definition proposed by a diabetes consultation panel for WHO in 1998 and finalized in 1999 [6–8]. MetS, a cluster of pathological conditions, is associated with a metabolic, proinflammatory, and prothrombotic state, which can increase the risk of atherogenic disorders [9]. Some studies have explored the association between vascular damage and MetS components. A review indicated that a complication of long-term obesity is vascular damage, which leads to early increases in arterial stiffness and accelerates the progression of atherosclerosis [10]. A study involving middle-aged participants found that higher systolic blood pressure categories were significantly association with PAD in a graded manner [11]. A cohort study indicated that elevated remnant cholesterol is linked to a five-fold increased risk of PAD in the general population [12]. A previous study in elderly patients with T2DM reported that the risk of PAD rises with the increase in the number of MetS components [13]. Another study in 8374 T2DM patients showed that MetS is specifically associated with an increased risk of lower extremity artery disease (LEAD) in female patients [14]. These studies suggest that age and gender are key factors influencing both MetS and PAD. Therefore, we aim to explore the association between PAD and MetS across different age and gender groups.

## 2. Material and Methods

### 2.1. Study Design and Population

From 2017 to 2021, we collected the clinical data from patients registered at the Endocrine Metabolism and Immune Diseases Center at Beijing Luhe Hospital, Capital Medical University. All patients participating in the National Metabolic Management Center (MMC) receive standardized health education and professional guidance on diabetes management.

This is a cross-sectional study. Patients with T2DM were enrolled, while those with other types of diabetes were excluded. T2DM is a chronic metabolic disease characterized by insulin resistance and relative insufficiency of insulin secretion. The diagnosis of T2DM is based on typical diabetes symptoms, including polydipsia, polyuria, polydipsia, and unexplained weight loss, and meets any of the following conditions: (1) random blood glucose ≥ 11.1 mmol/L, (2) fasting blood glucose (FBG) ≥ 7.0 mmol/L, (3) 2-h blood glucose levels ≥ 11.1 mmol/L during an oral glucose tolerance test (OGTT), and (4) glycosylated hemoglobin (HbA1c) ≥ 6.5%. If typical symptoms are absent, the patient must undergo testing on different days, and the results must meet any of the above criteria to confirm the diagnosis [15].

The study protocol was developed in accordance with the Declaration of Helsinki and approved by the Ethical Review Committee of Beijing Luhe Hospital (approval number: 2023-LHKY-094-02). Written informed consents were obtained from all participants involved in the study.

### 2.2. Data Collection

Data collection was conducted following the standard protocol of MMC [16, 17]. Baseline data, including age, gender, smoking and alcohol histories, duration of T2DM, and detailed medical history were recorded using a standard questionnaire. Weight, height, and waist circumference were measured by a trained professional. Visceral fat area (VFA) and subcutaneous fat area (SFA) were also assessed. Blood samples were collected after an overnight fast on the morning of the examination day. FBG, uric acid (UA), serum triglyceride (TG), total cholesterol (TC), low-density lipoprotein (LDL-C), and high-density lipoprotein (HDL-C) were measured using an automated biochemical analyzer (Roche/Hitachi Cobas C501, Roche Diagnostic Corp., Indianapolis). HbA1c concentrations were determined by high-performance liquid chromatography (HPLC) using a D10 set (Bio-RAD, Hercules, California). The estimated glomerular filtration rate (eGFR) was calculated using the Modification of Diet in Renal Disease (MDRD) equation.

 $$\begin{array}{c} \text{GFR}=186*{\left(\frac{\text{SCr}}{88.402}\right)}^{-1.154}*{\text{age}}^{-0.203}*\left(\text{women}*0.742\right) \end{array}$$

### 2.3. Measurement of ABI and Definition of PAD

Participants rested in a supine position for at least 5 min before the ankle–brachial index (ABI) was noninvasively measured using an automated recorder (BP-203RPE III, from PWV/ABI, Omron Healthcare Co.). An ABI value of ≤ 0.9 in either the right or left leg was defined as PAD [18], while ABI values > 0.9 in both legs were defined as NPAD.

### 2.4. Definition of MetS

Subjects with three or more of the following attributes were defined as having MetS: (a) waist circumference ≥ 90 cm for males and ≥ 85 cm for females; (b) fasting blood glucose ≥ 6.1 mmol/L or 2-h blood glucose ≥ 7.8 mmol/L after a glucose load or a diagnosis and treatment of diabetes; (c) blood pressure ≥ 130/85 mmHg (1 mmHg = 0.133 kPa) or a confirmed and treated diagnosis of hypertension; (d) fasting TG ≥ 1.70 mmol/L; (e) fasting HDL − C < 1.04 mmol/L [15]. Based on the number of MetS components, patients were grouped into three categories: *n* = 3, *n* = 4, and *n* = 5 components. Subjects with one or two of the above attributes, or none, were classified as non-MetS (NMetS).

### 2.5. Statistical Analysis

Statistical analyses were performed using SPSS 26.0 software. Continuous variables with normal distribution were presented as mean ± standard deviation, while skewed variables were presented as median (interquartile range, 25%–75%). Differences between the two groups were assessed using the independent-sample *t*-test or chi-square tests. Multiple logistic regression analyses were conducted to identify risk factors for patients with PAD and without PAD. Odds ratio and 95% confidence intervals were calculated, with potential confounding factors adjusted for. A *p* value < 0.05 was considered statistically significant.

## 3. Results

### 3.1. Baseline Clinical Parameters of the Study Patients

**Figure 1:**
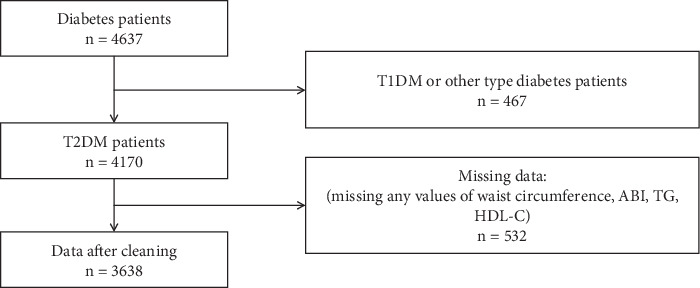
Flow chart.

In the cross-sectional study, patients with T2DM were enrolled, while those with other types of diabetes were excluded. Records with missing data for waist circumference, ABI, TG, or HDL-C were excluded from the analysis. Finally, a total of 3638 patients were enrolled, including 281 patients with PAD and 3357 patients without PAD (Figure 1).

Table 1 shows the demographic characteristics and laboratory data of patients with PAD and NPAD. Compared to NPAD patients, those with PAD were younger (49.3 vs. 52.2, *p* < 0.001) but had higher HbA1c, UA, TG, and hsCRP and lower HDL-C (all *p* < 0.01). Nonsignificant differences were observed between PAD and NPAD patients in terms of the duration of T2DM, FBG, TC, and LDL-C. BMI, waist circumference, VFA, and SFA were higher in PAD patients (all *p* < 0.01). The prevalence of MetS was higher in the PAD group compared to the NPAD group (86.1% vs. 76.0%, *p* < 0.001).

Given the different prevalence rates of PAD in male and female patients, we further explored the metabolic characteristics by gender (Supporting Information (available here)). Consistent with the findings for all patients, both male and female patients with PAD showed higher values in physical measurement indices, including BMI, waist circumference, VFA, and SFA (all *p* < 0.01). In both male and female PAD patients, HbA1c, UA, TG, and hsCRP levels were elevated, while HDL-C levels were lower (all *p* < 0.01). The incidence of MetS in the PAD group was statistically higher than that in the NPAD group. Female patients with PAD were younger than those in the NPAD group (51.9 vs. 55.4, *p* = 0.041), while no significant age difference was found in male patients (48.0 vs. 49.7, *p* = 0.152).

### 3.2. The Association Between MetS and the Presence of PAD

To further investigate the relationship between PAD and MetS, patients with PAD were divided into four groups based on the number of MetS components. Multiple logistic regression was used to assess the independent factors associated with PAD (Table 2). Variables showing significant differences between the PAD and NPAD groups in Table 1 were considered potential confounding factors. Before conducting the multivariate analysis, collinearity among the independent variables was assessed. Variables with significant collinearity were excluded, and the remaining variables were included in the multivariate analysis.

After adjusting for potential confounders, including age, duration of T2DM, HbA1c, DBP, smoking, UA, and hsCRP, our results showed that T2DM patients with MetS had a significantly higher prevalence of PAD (OR = 1.97, *p* = 0.001) compared to those with NMetS. The ORs increased with the number of MetS components. Patients with three MetS components had a 1.65 times greater risk of PAD compared to T2DM patients with NMetS. In addition, the OR increased to 2.0 and 2.73 for patients with four and five MetS components, respectively. Our findings also indicated that gender is an independent risk factor for PAD, with males (OR = 1.38, *p* = 0.045) having a higher risk of PAD than females after adjusting for confounders. Furthermore, we examined the relationship between various components of MetS and PAD (Table 3). After adjusting for relevant confounding factors, we found that hypertension (*p* = 0.001) and hyperlipidemia (*p* = 0.018) remained significant risk factors for PAD.

### 3.3. Stratified Analysis of the Association Between MetS and PAD

**Figure 2:**
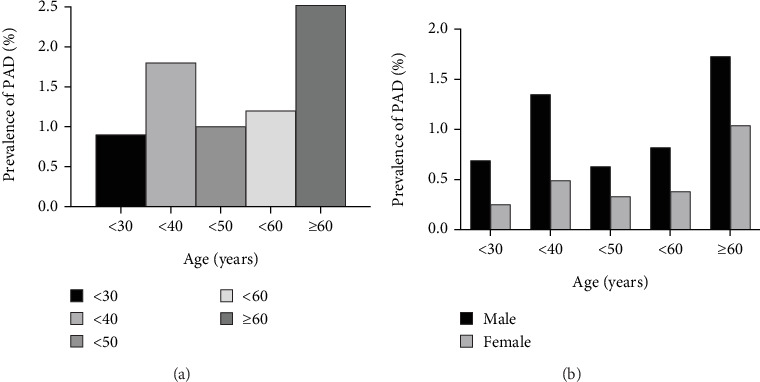
The prevalence of PAD. (a) Prevalence of PAD within the increased age. (b) Prevalence of PAD within the increased age in different genders. PAD, peripheral artery disease.

To further investigate the relationship between MetS and PAD, we performed stratified analysis by age and gender.  Figure 2 shows that patients aged 30–40 years and ≥ 60 years had a higher incidence of PAD. Therefore, we divided the patients into three groups: aged < 40 years, 40–59 years, and ≥ 60 years. Stratified analyses showed that adjusted ORs increased with the number of MetS components in both younger (aged < 40 years) and older patients (aged ≥ 60 years) patients. In elderly patients, the adjusted ORs for PAD were 1.61 (*p* = 0.191), 2.55 (*p* = 0.012), and 3.20 (*p* = 0.015) for three, four, and five MetS components, respectively, after adjusting for confounding factors. In younger patients, those with three MetS components had a 1.59 times greater risk of PAD than patients with NMetS, and the OR increased to 2.04 and 3.27 for patients with four and five MetS components, respectively. However, no positive association between the number of MetS components and the presence of PAD was found in middle-aged patients (40 years ≤ age < 60 years) (Table 4).

Next, we conducted a gender subgroup analysis to explore the relationship between MetS components and the presence of PAD (Table 5). In females with three, four, and five MetS components, the adjusted ORs for PAD were 2.46 (*p* = 0.029), 2.94 (*p* = 0.012), and 6.28 (*p* < 0.001), respectively. Similarly, the adjusted ORs in males increased with the number of MetS components, but there was no statistical significance for males with three components of MetS (OR = 1.45, *p* = 0.189).

## 4. Discussion

MetS is an independent risk factor for PAD. Our study suggested that patients with MetS are more likely to develop PAD, and the risk of PAD positively correlated with the number of MetS components. One study, which included 1958 participants from the Northern Shanghai Study aged over 65 years without a history of cardiovascular disease, showed that patients with more MetS components had lower ABI values [19]. Another study with 1592 participants ages 55–74 years found that a lower ABI was more prevalent among individuals with MetS compared to those without [20]. Moreover, some studies have explored the association between MetS and PAD. A multiethnic study revealed that people free of clinical cardiovascular disease with MetS are at increased risk for PAD [21]. Another study indicated that endothelial dysfunction partly explained the association between MetS and the severity of PAD [22]. A previous study in elderly patients with T2DM reported that the risk of PAD increases with the number of MetS components [13]. Unlike this study, our study explored the relationship between PAD and the number of MetS components across different groups categorized by age and gender. We found that the prevalence of PAD was high in older patients (≥ 60 years), which may be attributed to the longer duration of T2DM among this group, as well as the high prevalence of chronic diseases such as hypertension and hyperlipidemia, which contribute to atherosclerosis development. Interestingly, we also observed a high occurrence of PAD in younger patients aged 30–40 years. An unhealthy lifestyle, marked by physical inactivity, poor dietary habits, and psychological stress from social factors, is strongly associated with the prevalence and mortality of atherosclerosis [23]. Research suggests that adopting a healthy lifestyle, such as limiting alcohol consumption, maintaining a balanced diet, and ensuring adequate sleep, can significantly reduce the risk of PAD [24, 25]. A 2018 study found an inverse relationship between age and weekly sedentary time, indicating that younger individuals tend to spend more hours sitting. Moreover, patients with T2DM who have prolonged sedentary periods face an increased risk of arterial plaque buildup [26]. Additionally, younger individuals often grapple with higher life pressures, shorter sleep durations, and other harmful lifestyle behaviors, potentially contributing to the rising incidence of PAD among those aged 30–40. This finding highlights the importance of paying closer attention to the incidence of MetS in younger patients, rather than focusing solely on the elderly patients.

In our study, we found that the risk of PAD was higher in the females with MetS than those without MetS. After menopause, the decline in estrogen levels makes females more susceptible to metabolic abnormalities such as hyperlipidemia, which in turn increases the risk of PAD. In addition, the obesity rate among female patients is higher than in males, and the insulin resistance and inflammatory response caused by obesity exacerbate arteriosclerosis, leading to a higher risk of PAD in females [27, 28]. Furthermore, we observed that the risk of PAD increased with the number of MetS components in females. A previous study involving 8374 T2DM patients showed that MetS is specifically associated with an increased risk of LEAD among female T2DM patients, but MetS may not be as significant in the prevalence of LEAD in male T2DM patients [14]. In the group of female patients with T2DM, we should focus not only on the blood glucose but also monitor for other chronic diseases, such as hypertension and hyperlipidemia. Although our findings are slightly different from this study, we also observed that male patients with MetS and four or five components have a progressively higher risk of developing PAD compared to those without MetS. After adjusting for potential confounding factors, the ORs were 1.75 and 1.95, respectively. These differences may be attributed to variations in the composition of MetS.

The present study has several limitations. First, this is a single-center study, which may limit the generalizability of the findings. Second, PAD was defined based on the ABI in the absence of clinical symptoms such as acral numbness or intermittent claudication of lower limbs. While we did perform vascular ultrasound to assess the condition of peripheral arteries in some patients, which may contribute to a more accurate diagnosis of PAD, the lack of widespread ultrasound use may have affected the overall assessment.

## 5. Conclusions

In summary, our results suggest that the risk of PAD is positively associated with the number of MetS components in patients with T2DM, especially in both younger patients and older patients. Additionally, the positive correlation between the number of MetS components and the presence of PAD was significantly more pronounced in female patients.

## Figures and Tables

**Table 1:** Baseline demographics characteristics and clinical laboratory data in patients.

	**PAD (** **n** = 281**)**	**NPAD (** **n** = 3357**)**	**p** ** value**
Age (year)	49.3 ± 16.0	52.2 ± 12.2	< 0.001
Gender			< 0.001
Male	190 (67.6%)	1883 (56.1%)	
Female	91 (32.4%)	1474 (43.9%)	
Anthropometric indices			
BMI (kg/cm^2^)	28.8 ± 4.7	26.7 ± 4.0	< 0.001
Waist circumference (cm)	99.0 ± 11.4	94.4 ± 10.5	< 0.001
VFA (cm^2^)	124.5 ± 49.5	104.7 ± 40.6	< 0.001
SFA (cm^2^)	231.6 ± 91.6	201.7 ± 67.1	< 0.001
SBP (mmHg)	132.0 ± 16.9	133.1 ± 17.9	0.316
DBP (mmHg)	78.3 ± 12.2	80.2 ± 11.8	0.012
Duration of T2DM (mon)	47 (1, 143)	64 (9, 137)	0.229
FBG (mmol/L)	8.2 (6.6, 11.5)	8.2 (6.6, 10.9)	0.914
HbA1c (%)	9.4 ± 2.3	8.9 ± 2.2	< 0.001
eGFR (mL/min/1.73m^2^)	106.8 ± 32.4	106.7 ± 32.0	0.984
UA (mmol/L)	357.7 ± 107.1	324.9 ± 89.1	< 0.001
TG (mmol/L)	1.76 (1.2, 2.7)	1.54 (1.1, 2.3)	< 0.001
TC (mmol/L)	4.9 ± 1.6	4.9 ± 1.3	0.615
HDL-C (mmol/L)	1.1 ± 0.3	1.2 ± 0.3	< 0.001
LDL-C (mmol/L)	3.2 ± 1.0	3.1 ± 0.9	0.378
hsCRP (mg/L)	2.3 (1.1, 4.9)	1.6 (0.8, 3.6)	< 0.001
Smoking, *n* (%)	140 (49.8%)	1387 (41.3%)	0.006
Drinking, *n* (%)	148 (52.7%)	1568 (46.7%)	0.056
MetS, *n* (%)	242 (86.1%)	2550 (76.0%)	< 0.001
Abdominal obecity, *n* (%)	232 (82.6%)	2588 (77.1%)	0.035
Hypertension, *n* (%)	205 (73.0%)	2344 (69.8%)	0.271
Hyperlipidemia, *n* (%)	209 (74.4%)	1909 (56.9%)	< 0.001
History of medicine, *n* (%)			
Hypoglycemic drugs, *n* (%)	198 (70.7%)	2422 (72.3%)	0.581
Lipid-lowering drugs, *n* (%)	71 (25.4%)	872 (26.0%)	0.816
Antihypertensive drugs, *n* (%)	99 (35.2%)	1211 (36.1%)	0.769

*Note:* Continuous variables are expressed as mean ± SD or median (interquartile ranges), while categorical are shown as numbers with percentages.

Abbreviations: BMI, body mass index; DBP, diastolic blood pressure; eGFR, estimated glomerular filtration rate; FBG, fasting blood glucose; HbA1c, glycosylated hemoglobin; HDL-C, high-density lipoprotein cholesterol; hsCRP, hypersensitive C reactive protein; LDL-C, low-density lipoprotein cholesterol; SBP, systolic blood pressure; SFA, subcutaneous fat area; TC, total cholesterol; TG, triglycerides; UA, uric acid; VFA, visceral fat area.

**Table 2:** The independent factors of PAD by multiple logistic regression.

	**Model 1** ^**a**^		**Model 2** ^**b**^		**Model 3** ^**c**^	
	**OR (95% CI)**	**p** ** value**	**OR (95% CI)**	**p** ** value**	**OR (95% CI)**	**p** ** value**
Gender	1.47 (1.12, 1.92)	0.005	1.59 (1.21, 2.09)	0.001	1.38 (1.01, 1.90)	0.045
MetS	1.92 (1.35, 2.72)	< 0.001	2.30 (1.61, 3.29)	< 0.001	1.97 (1.30, 2.98)	0.001
NMetS	—	—	—	—	—	—
MetS (*n* = 3)	1.57 (1.06, 2.31)	0.025	1.87 (1.25, 2.78)	0.002	1.65 (1.05, 2.59)	0.030
MetS (*n* = 4)	2.01 (1.37, 2.94)	< 0.001	2.42 (1.63, 3.58)	< 0.001	2.00 (1.27, 3.15)	0.003
MetS (*n* = 5)	2.73 (1.76, 4.24)	< 0.001	3.47 (2.21, 5.47)	< 0.001	2.73 (1.59, 4.66)	< 0.001

*Note:* Values are presented as odds ratio (95% confidence interval). MetS (*n* = 3): the number of MetS components is three. MetS (*n* = 4): the number of MetS components is four. MetS (*n* = 5): the number of MetS components is five. NMetS: without MetS.

Abbreviations: CI, confidence interval; OR, odds ratio; PAD, peripheral artery disease.

^a^Model 1 was adjusted for age and duration of T2DM.

^b^Model 2 was adjusted for age, duration of T2DM, HbA1c, DBP, and smoking.

^c^Model 3 was controlled for age, duration of T2DM, HbA1c, DBP, smoking, UA, and hsCRP.

**Table 3:** Multivariate logistic regression analysis of the association of MetS components and PAD.

	**Model 1** ^**a**^		**Model 2** ^**b**^		**Model 3** ^**c**^	
	**OR (95% CI)**	**p** ** value**	**OR (95% CI)**	**p** ** value**	**OR (95% CI)**	**p** ** value**
Abdominal obecity	1.23 (0.89, 1.71)	0.216	1.32 (0.94, 1.83)	0.106	1.12 (0.77, 1.64)	0.541
Hypertension	1.17 (0.88, 1.55)	0.276	1.75 (1.28, 2.41)	< 0.001	1.83 (1.26, 2.66)	0.001
Hyperlipidemia	1.92 (1.44, 2.56)	< 0.001	1.83 (1.37, 2.44)	< 0.001	1.49 (1.07, 2.07)	0.018

*Note:* Values are presented as odds ratio (95% confidence interval).

^a^Model 1 was adjusted for gender, age, and duration of T2DM.

^b^Model 2 was adjusted for gender, age, duration of T2DM, HbA1c, DBP, and smoking.

^c^Model 3 was controlled for gender, age, duration of T2DM, HbA1c, DBP, smoking, UA, and hsCRP.

**Table 4:** Age subgroup analysis of the association between PAD and the number of components of MetS.

	**Model 1** ^**a**^		**Model 2** ^**b**^		**Model 3** ^**c**^	
	**OR (95% CI)**	**p** ** value**	**OR (95% CI)**	**p** ** value**	**OR (95% CI)**	**p** ** value**
Age < 40						
NMetS	—	—	—	—	—	—
MetS (*n* = 3)	1.45 (0.68, 3.09)	0.333	1.75 (0.81, 3.77)	0.154	1.59 (0.69, 3.68)	0.279
MetS (*n* = 4)	2.11 (1.07, 4.16)	0.032	2.63 (1.29, 5.36)	0.008	2.04 (0.93, 4.50)	0.076
MetS (*n* = 5)	3.17 (1.53, 6.56)	0.002	4.25 (1.93, 9.34)	< 0.001	3.27 (1.37, 7.83)	0.008

40 ≤ age < 60						
NMetS						
MetS (*n* = 3)	2.332 (1.09, 5.0)	0.029	2.39 (1.11, 5.13)	0.025	2.13 (0.90, 5.09)	0.087
MetS (*n* = 4)	2.67 (1.26, 5.68)	0.010	2.55 (1.20, 5.44)	0.015	2.13 (0.89, 5.10)	0.091
MetS (*n* = 5)	3.30 (1.34, 8.12)	0.009	3.29 (1.34, 8.11)	0.010	2.30 (0.80, 6.63)	0.124

Age ≥ 60						
NMetS						
MetS (*n* = 3)	1.25 (0.69, 2.26)	0.468	1.56 (0.85, 2.87)	0.149	1.61 (0.79, 3.30)	0.191
MetS (*n* = 4)	1.79 (0.97, 3.29)	0.062	2.25 (1.21, 4.20)	0.011	2.55 (1.23, 5.30)	0.012
MetS (*n* = 5)	1.84 (0.86, 3.94)	0.118	2.28 (1.05, 4.95)	0.037	3.20 (1.25, 8.17)	0.015

*Note:* Values are presented as odds ratio (95% confidence interval). MetS (*n* = 3): the number of MetS components is three. MetS (*n* = 4): the number of MetS components is four. MetS (*n* = 5): the number of MetS components is five. NMetS: without MetS.

Abbreviations: CI, confidence interval; OR, odds ratio; PAD, peripheral artery disease.

^a^Model 1 was adjusted for gender and duration of T2DM.

^b^Model 2 was adjusted for gender, duration of T2DM, HbA1c, DBP, and smoking.

^c^Model 3 was controlled for gender, duration of T2DM, HbA1c, DBP, smoking, UA, and hsCRP.

**Table 5:** Subgroup analysis of the association between PAD and the number of components of MetS for gender.

	**Model 1** ^**a**^		**Model 2** ^**b**^		**Model 3** ^**c**^	
	**OR (95% CI)**	**p** ** value**	**OR (95% CI)**	**p** ** value**	**OR (95% CI)**	**p** ** value**
*Male*						
NMetS	—	—	—	—	—	—
MetS (*n* = 3)	1.28 (0.80, 2.07)	0.305	1.53 (0.94, 2.49)	0.088	1.45 (0.83, 2.53)	0.189
MetS (*n* = 4)	1.77 (1.13, 2.77)	0.013	2.12 (1.33, 3.38)	0.002	1.75 (1.01, 3.03)	0.045
MetS (*n* = 5)	2.02 (1.20, 3.39)	0.008	2.51 (1.45, 4.33)	0.001	1.95 (1.01, 3.75)	0.045

*Female*						
NMetS	—	—	—	—	—	—
MetS (*n* = 3)	2.31 (1.15, 4.62)	0.019	2.79 (1.37, 5.67)	0.005	2.46 (1.10, 5.52)	0.029
MetS (*n* = 4)	2.76 (1.36, 5.62)	0.005	3.36 (1.62, 6.96)	0.001	2.94 (1.27, 6.80)	0.012
MetS (*n* = 5)	6.77 (3.05, 15.04)	< 0.001	8.55 (3.75, 19.48)	< 0.001	6.28 (2.33, 16.97)	< 0.001

*Note:* Values are presented as odds ratio (95% confidence interval). MetS (*n* = 3): the number of MetS components is three. MetS (*n* = 4): the number of MetS components is four. MetS (*n* = 5): the number of MetS components is five. NMetS: without MetS.

Abbreviations: CI, confidence interval; OR, odds ratio; PAD, peripheral artery disease.

^a^Model 1 was adjusted for age and duration of T2DM.

^b^Model 2 was adjusted for age, duration of T2DM, HbA1c, DBP, and smoking.

^c^Model 3 was controlled for age, duration of T2DM, HbA1c, DBP, smoking, UA, and hsCRP.

## Data Availability

The data that support the findings of this study are available from the corresponding authors upon reasonable request.

## Supporting Information

Supporting Information Additional supporting information can be found online in the Supporting Information section. A comparison of baseline demographics characteristics and clinical laboratory data by gender (Table S1) is shown in the supporting information.
