# Supplementary material for: Age and Gender Difference in the Association of Metabolic Syndrome and Peripheral Artery Disease Among Patients With Type 2 Diabetes Mellitus
Source: Int J Vasc Med. 2025 Mar 10;2025:5572344. doi: 10.1155/ijvm/5572344 (PMC11991799; doi:10.1155/ijvm/5572344)
Supplement: Supporting Information — Additional supporting information can be found online in the Supporting Information section. A comparison of baseline demographics characteristics and clinical laboratory data by gender (Table S1) is shown in the supporting information. [file 5572344.f1.docx]

**Supplementary Table 1** Comparison of baseline demographics characteristics and clinical laboratory data in male group and female group.

|  | Male (n = 2073) | |  | Female (n = 1565) | |  |
| --- | --- | --- | --- | --- | --- | --- |
|  | PAD | NPAD | *P* value | PAD | NPAD | *P* value |
| Age (year)  Anthropometric indices  BMI (kg/cm^2^)  Waist circumference (cm)  Hip circumference (cm)  VFA (cm^2^)  SFA (cm^2^)  SBP (mmHg)  DBP (mmHg)  Duration of T2DM (mon)  FBG (mmol/l)  HbA1c (%)  eGFR (ml/min/1.73m^2^)  UA (mmol/l)  TG (mmol/l)  TC (mmol/l)  HDL-C (mmol/l)  LDL-C (mmol/l)  hsCRP (mg/l)  Smoking, n (%)  Drinking, n (%)  MetS, n (%)  Abdominal obecity, n (%)  Hypertension, n (%)  Hyperlipidemia, n (%)  History of medicine, n (%)  Hypoglycemic drugs, n (%)  Lipid-lowering drugs, n (%)  Antihypertensive drugs, n (%) | 48.0 ± 15.9  28.2 ± 4.2  99.2 ± 10.8  103.5 ± 8.7  125.8 ± 46.2  222.8 ± 83.1  131.8 ± 17.4  79.7 ± 11.6  43(0, 125.3)  9.5 ± 4.5  9.4 ± 2.3  108.2 ± 31.8  371.9 ± 106.4  1.8 (1.2, 2.8)  4.9 ± 1.6  1.1 ± 0.3  3.2 ± 1.0  2.1 (1.1, 4.5)  135(71.1)  139(73.2)  162 (85.3)  154 (81.1)  138 (72.6)  145 (76.3)  131 (69.3)  49 (25.9)  61 (32.1) | 49.7 ± 12.0  27.0 ± 3.9  96.4 ± 10.1  101.5 ± 7.7  113.1 ± 42.2  201.6 ± 67.2  132.8 ± 17.7  82.1 ± 12.0  49(3, 128)  9.6 ± 4.3  8.9 ± 2.3  107.9 ± 31.4  344.6 ± 87.4  1.6(1.1, 2.5)  4.8 ± 1.3  1.1 ± 0.3  3.1 ± 0.9  1.5 (0.7, 3.3)  1280(68.0)  1391(73.9)  1470 (78.1)  1466 (77.9)  1307 (69.4)  1187 (63.0)  1297 (69.0)  452 (24.0)  605 (32.2) | 0.152  < 0.001  < 0.001  0.002  < 0.001  0.001  0.436  0.009  0.442  0.674  0.006  0.882  0.001  0.019  0.267  0.026  0.204  0.001  0.385  0.831  0.021  0.309  0.357  < 0.001  0.935  0.526  0.987 | 51.9 ± 15.9  30.0 ± 5.5  98.4 ± 12.6  106.2 ± 11.2  121.9 ± 55.9  250.2 ± 105.4  132.4 ± 16.0  75.3 ± 13.0  63(8, 197)  9.3 ± 3.5  9.5 ± 2.3  103.7 ± 33.5  328.4 ± 103.0  1.8(1.2, 2.5)  4.9 ± 1.4  1.1 ± 0.3  3.1 ± 1.1  3.1 (1.2, 6.4)  5(5.5)  9(9.9)  80 (87.9)  78 (85.7)  67 (73.6)  64 (70.3)  67 (73.6)  22 (24.2)  38 (41.8) | 55.4 ± 11.6  26.4 ± 4.0  91.9 ± 10.5  100.1 ± 8.5  93.9 ± 35.8  201.8 ± 67.0  133.4 ± 18.1  77.7 ± 11.2  84(21, 157.5)  9.1 ± 3.9  8.8 ± 2.1  105.2 ± 32.7  299.5 ± 84.8  1.5(1.0, 2.1)  5.0 ± 1.3  1.3 ± 0.3  3.1 ± 1.0  1.8 (0.8, 4.0)  107(7.3)  177(12.0)  1080 (73.3)  1122 (76.1)  1037 (70.4)  722 (49.0)  1125 (76.4)  420 (28.5)  606 (41.1) | 0.041  < 0.001  < 0.001  < 0.001  < 0.001  < 0.001  0.603  0.055  0.968  0.706  0.002  0.671  0.01  0.008  0.661  < 0.001  0.929  0.002  0.524  0.542  0.002  0.036  0.506  < 0.001  0.550  0.375  0.907 |

Abbreviations: BMI, body mass index; DBP, diastolic blood pressure; eGFR, estimated glomerular filtration rate; FBG, fasting blood glucose; HbA1c, glycosylated hemoglobin; HDL-C, high-density lipoprotein cholesterol; hsCRP, hypersensitive C Reactive Protein; LDL-C, low-density lipoprotein cholesterol; SBP, systolic blood pressure; SFA, subcutaneous fat area; TC, total cholesterol; TG, triglycerides; UA, uric acid; VFA, visceral abdominal fat area.
